# Combined standard immunosuppression and immune checkpoint inhibition for BKPyV+ metastatic renal cell carcinoma of the graft in a kidney transplant recipient with chronic rejection: a case report

**DOI:** 10.3389/fonc.2025.1506324

**Published:** 2025-03-17

**Authors:** Ilaria Gandolfini, Martina Manini, Giuseppe Daniele Benigno, Micaela Gentile, Alessandra Palmisano, Danio Somenzi, Letizia Gnetti, Marco Delsante, Benedetta Mordà, Marta D’Angelo, Daniel Salvetti, Enrico Fiaccadori, Sebastiano Buti, Umberto Maggiore

**Affiliations:** ^1^ UO Nefrologia, Azienda Ospedaliero-Universitaria di Parma, Parma, Italy; ^2^ UO Oncologia Medica, Dipartimento di Medicina e Chirurgia, Università di Parma, Parma, Italy; ^3^ Unit (UO) Nefrologia e Dialisi, Azienda USL-IRCCS Reggio Emilia, Reggio Emilia, Italy; ^4^ UO Anatomia Patologica, Azienda Ospedaliero-Universitaria di Parma, Parma, Italy; ^5^ Dipartimento di Medicina e Chirurgia, Università di Parma, Parma, Italy

**Keywords:** immune checkpoint inhibitors, immunosuppression, BK polyomavirus, renal cell carcinoma, kidney transplantation, dialysis

## Abstract

We report on the first case of a dual-kidney transplant recipient diagnosed with a metastatic BK polyomavirus-positive clear renal cell carcinoma with sarcomatoid features, which caused extensive vena cava thrombosis. The patient was successfully treated with the immune checkpoint inhibitors (ICIs) ipilimumab plus nivolumab and continued immunosuppression with tacrolimus, mycophenolate, and steroids. He received ICIs despite the presence of graft dysfunction due to transplant glomerulopathy. As expected, the ICI treatment caused a progressive but asymptomatic decline of the graft function, which resulted in end-stage kidney disease. However, continuation of a full immunosuppression prevented acute rejection, graft intolerance syndrome episodes, or dual graft nephrectomy, which enabled the patient to successfully continue ICIs while on dialysis and to achieve sustained partial remission at the 17-month follow-up.

## Introduction

The management of carcinoma of the kidney graft poses several challenges, especially in the era of immune checkpoint inhibitors (ICIs) (1, 2). Among the several ICI combinations available for first-line treatment of metastatic renal cell carcinoma, ipilimumab plus nivolumab is an option for patients with dismal prognosis. In fact, as shown by the CheckMate 214 phase III trial, the overall survival and objective response rates were significantly higher with nivolumab plus ipilimumab than with sunitinib among intermediate- and poor-risk patients with previously untreated renal cell carcinoma (3).

ICIs, by unleashing the T-cell immunity against cancer cells, increase the risk of graft rejection, potentially leading to graft loss (1, 4, 5). As transplant physicians often reduce immunosuppression to promote ICI-driven cancer clearance, the incidence of severe acute rejection and the subsequent ICI discontinuation have been as high as 40% in kidney transplant recipients (5, 6). On the contrary, two recent small phase 1 clinical trials (in 17 and 12 patients, respectively) have shown that, by continuing immunosuppressive (IS) therapy, the incidence of acute rejection can be kept well below 15%, without apparently compromising the anticancer response (4, 7). These findings were, however, not confirmed by another small trial in eight patients, which showed that tacrolimus and prednisone are insufficient to prevent graft loss and can compromise the ICI immune-mediated tumor regression (8). In all three trials, ICIs were discontinued in patients who developed acute rejection. Avoiding acute rejection and/or graft intolerance syndrome is essential to enable the continuation of ICIs. Moreover, the anti-rejection treatment, which is based on pulse steroids, T-cell-depleting agents, and plasmapheresis, can nullify the action of ICIs (1, 2). While treatment of acute rejection is mandatory to limit the risk of life-threatening complications, the same does not apply to chronic rejection, which causes a slow and an asymptomatic decrease of graft function. One possible strategy to overcome the problem of acute rejection is to continue the standard maintenance immunosuppression at the time of ICI initiation. Would a severe rejection develop regardless, graft nephrectomy might be considered as an additional therapy that may enable the continuation of ICIs even in patients with uncontrolled acute rejection. Indeed, in kidney transplant patients who develop chronic rejection and eventually start dialysis, ICIs can be continued indefinitely (9, 10). It has also been argued that BK polyomavirus (BKPyV)-associated urothelial and kidney cancer may be especially sensitive to immune reconstitution in kidney transplant recipients; therefore, ICIs could be particularly effective in this setting (11).

Here, we report on the first case of a BKPyV-positive metastatic clear cell renal carcinoma that developed in the kidney graft in the context of chronic rejection, which was treated with the combination of ipilimumab plus nivolumab.

## Case description

A 57-year-old ever smoker man, with a history of hypertension, underwent a bilateral dual-kidney transplantation for end-stage renal disease secondary to IgA nephropathy. He received thymoglobulin induction and maintenance therapy with tacrolimus, everolimus, and steroids. His transplant history was uneventful, apart from a persistent urinary BKPyV DNA. However, 10 years post-transplant, after steroid self-withdrawal, his kidney function worsened (s-creatinine from 1 to 2.6 mg/dL; proteinuria, 2 g/day). Graft biopsy showed transplant glomerulopathy with microvascular inflammation, suggestive of chronic rejection, with no circulating anti-human leukocyte antigen (HLA) donor-specific antibodies (DSAs) or peritubular capillary C4d deposition. After replacing everolimus with mycophenolate and restarting low-dose steroids, his kidney function and proteinuria improved (s-creatinine, 1.9 mg/dl; proteinuria, 0.3 g/day).

**Figure 1:**
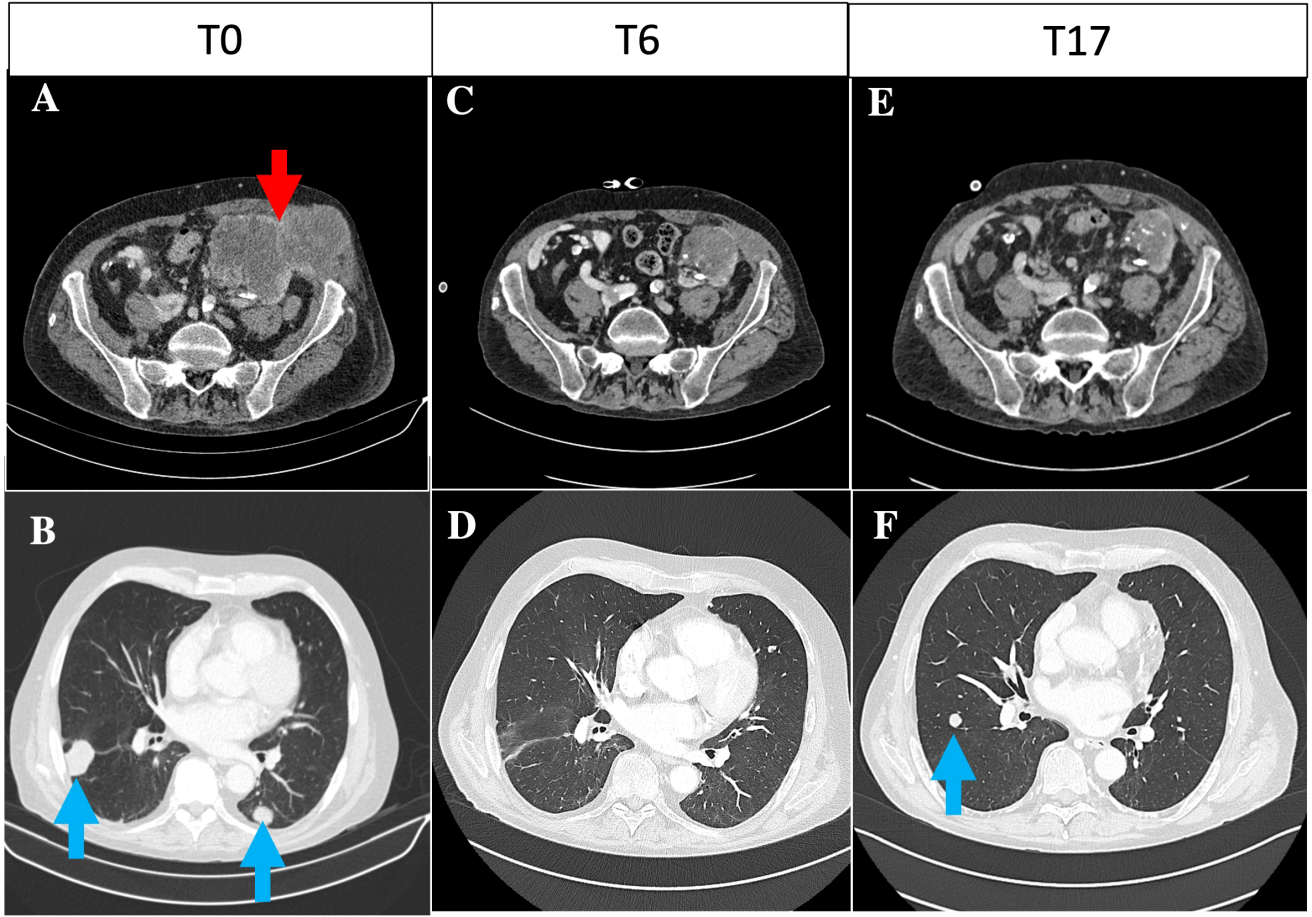
**(A, B)** Basal contrast-enhanced CT of the pelvis and thorax at baseline. **(A)** Lobulated contrast-enhanced expansive lesion of approximately 12 cm in the left graft with extension in the context of the abdominal wall muscles (71 × 63 mm, *red arrow*). **(B)** Multiple parenchymal nodulations in both lungs suggestive for metastases, the largest of approximately 18 mm in the right lower lobe (*blue arrows*). **(C, D)** Contrast-enhanced CT of the pelvis and thorax at baseline 6 months post-immune checkpoint inhibitor (ICI) initiation. **(C)** Lobulated expansive lesion in the left graft (50 × 46 mm), with extension in the context of the abdominal wall muscles (42 × 22 mm). **(D)** Overall reduction of multiple parenchymal nodulations in both lungs. **(E, F)** Contrast-enhanced CT of the pelvis and thorax at baseline 17 months post-ICI initiation. **(E)** Stable lobulated expansive lesion in the left graft (43 × 39 mm), with extension in the context of the abdominal wall muscles (40 × 18 mm). **(F)** Overall lung progression, with increase of some pulmonary nodules, the largest of 14 mm at the upper left lobe and 15 mm at the right lower lobe.

**Figure 2:**
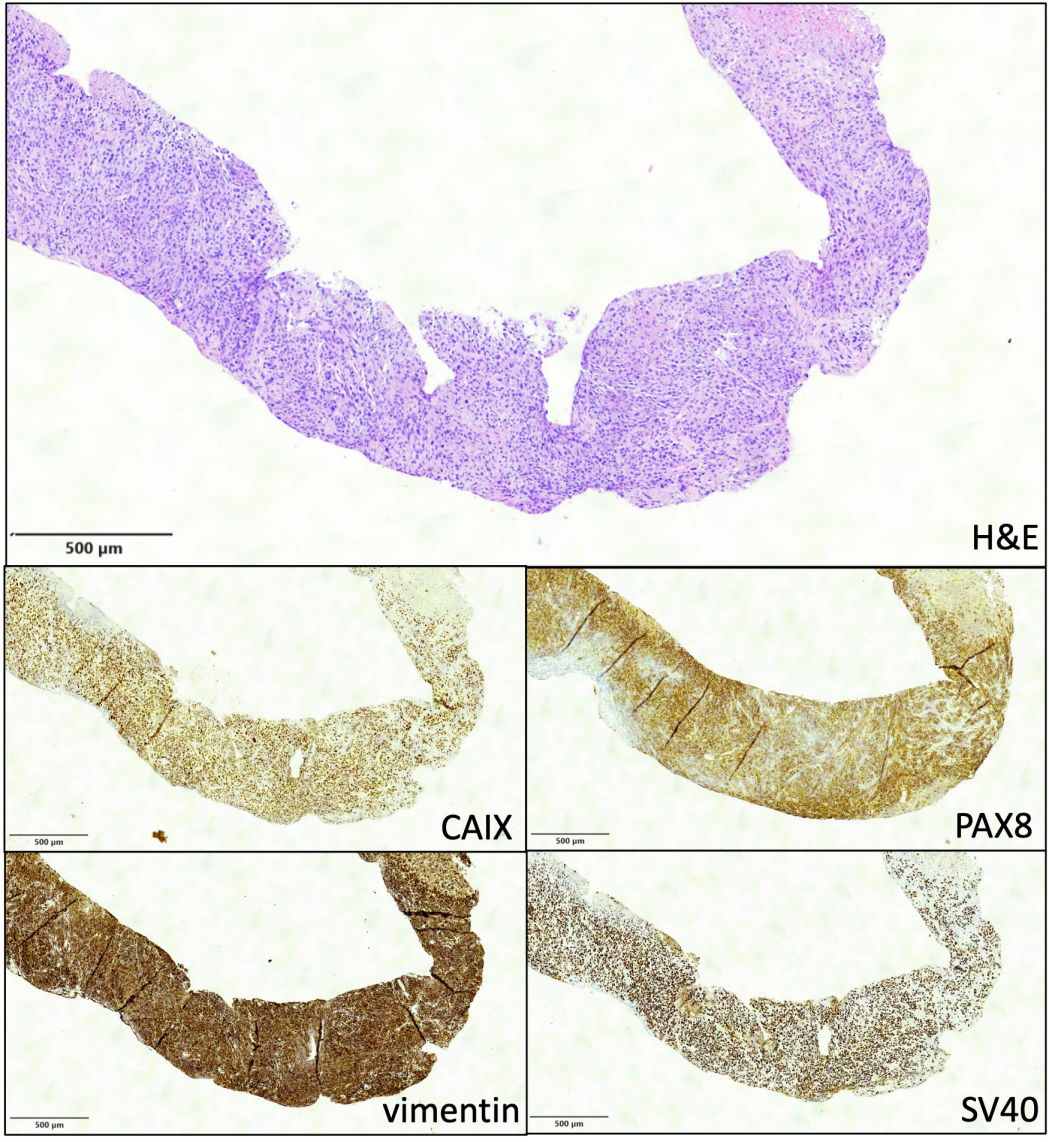
Needle biopsy of the renal neoplasm (×4). *Upper panel* shows an undifferentiated carcinoma at hematoxylin and eosin stain (×4). Immunohistochemistry (IHC) was overall compatible with a clear cell carcinoma with sarcomatoid differentiation staining positive for CAIX, PAX8, and vimentin. Diffuse nuclear positivity for BKPyV SV40 by IHC was detected in neoplastic cells, while the surrounding healthy kidney tissue was negative.

After 3 years, at the age of 70, the patient presented with a painful mass at the left iliac fossa, which was associated with a further deterioration of kidney function (s-creatinine, 2.6 mg/dl). A CT scan showed a 12-cm lobulated mass of the left kidney graft, with local abdominal wall and iliac muscle extension, multiple parenchymal nodulations in both lungs, and neoplastic thrombosis in the inferior vena cava, which required anticoagulation treatment (**Figures 1A, B**). The biopsy of the lesion showed clear cell carcinoma with sarcomatoid differentiation that stained positive for SV40-BKPyV in the nuclei, with the surrounding healthy kidney tissue being negative, and positive for the sarcomatoid/clear cell markers carbonic anhydrase IX (CAIX), paired box gene 8 (PAX8), and vimentin (**Figure 2**). Moreover, the brain CT scan and MRI showed multiple brain metastases.

The multidisciplinary team shared the treatment decision with the patient: due to the dismal prognosis related to disease extension, the poor IMDC (International Metastatic RCC Database Consortium) score, prognostic group 4 according to the Meet-URO score, and the sarcomatoid features (12, 13), we opted for first-line treatment with the combination of ipilimumab plus nivolumab despite the history of chronic rejection and the lack of available data in similar settings. The patient accepted the high risk of graft loss due to rejection and the option of urgent dual graft nephrectomy would a severe acute rejection develop (1).

To minimize the risk of acute rejection during the use of ICIs, and in accordance with the results of recent phase 1 trials (4, 7), the patient was maintained on IS therapy with tacrolimus, mycophenolate mofetil, and methylprednisolone 4 mg daily.

Following the first administration of ICIs, the kidney function progressively worsened, from 2.6 to 4 mg/dl over the course of 4 weeks. This was associated with an increase of urinary chemokine CXCL9 (from 123 to 342 pg/ml), which suggested ongoing chronic rejection. At 8 weeks after starting ICI therapy, the patient became dialysis-dependent (two dialysis sessions per week). Nonetheless, the graft biopsy documented only persistent chronic lesions, without signs of acute inflammation, and circulating anti-HLA DSAs remained negative.

The ICI treatment was well tolerated. The patient achieved partial remission at 6 months post-ICI initiation (**Figures 1C, D**): the palpable abdominal mass showed a remarkable reduction, and the performance status improved [European Cooperative Oncology Group Performance Score (ECOG PS) from 3 to 1] (14). Ipilimumab was withdrawn, and nivolumab was continued as maintenance ICI. The 9-month CT scan documented a further decrease of the abdominal mass; however, three lung nodules reappeared, suggesting a condition of “oligoprogression” (not shown). Nonetheless, the patient maintained a good performance status (ECOG PS = 1) without symptoms related to the cancer, rejection, or graft intolerance syndrome. No BKPyV DNAemia was detected during the entire follow-up. While on nivolumab, the patient was treated with stereotactic body radiotherapy on the three pulmonary nodules (month 15). The last brain MRI (15 months after ICI initiation) showed a further response of the brain metastases (2 *vs*. 15 lesions), and the 17-month CT scan confirmed that he maintained partial response (**Figures 1E, F**).

## Discussion

Our case presents a challenging situation in which a dual-kidney transplant recipient aged 70 years, with a baseline graft dysfunction secondary to chronic rejection, was diagnosed with a BKPyV-positive clear cell renal carcinoma with sarcomatoid features of the left kidney graft with lung and brain metastases.

The extension of the disease, the presence of a contralateral graft, and the bad performance status of the patient contraindicated the surgery-first approach. For this reason, we opted for a first-line treatment with a combination of ICIs (ipilimumab and nivolumab), as recent data on non-transplanted individuals have suggested a better response when compared with traditional targeted therapies (15).

The patient was informed by our multidisciplinary team about the concrete risk of acute rejection requiring urgent dual nephrectomy and anti-rejection treatment, which could have nullified the effect of the life-saving treatment with ICIs (1, 2).

To mitigate the risk of acute rejection, which was particularly high in our patient due to ongoing chronic rejection (6), we continued a full maintenance immunosuppression with tacrolimus, mycophenolate, and steroids, as suggested in recent phase 1 trials (4, 7).

As expected, the kidney function progressively worsened for the ongoing chronic rejection. Nonetheless, the patient never developed acute rejection or graft intolerance syndrome; therefore, he did not require additional IS treatment nor dangerous urgent dual nephrectomy. Rather, the patient could continue ICIs without interruption, even after starting chronic dialysis for a slow and asymptomatic deterioration of graft function (9, 10).

More importantly, this approach did not compromise the anticancer response; therefore, the patient achieved a sustained partial remission after 17 months of follow-up, with an improvement of his performance status.

To the best of our knowledge, no similar cases treated with the combination of ipilimumab and nivolumab have been reported in the literature to date. We contend that the strong positivity for BKPyV on cancer cell nuclei further supported the rationale of the use of ICI treatment (16).

Our case suggests that BKPyV-positive metastatic renal cell carcinoma of the kidney graft with sarcomatoid features can be successfully treated with ipilimumab and nivolumab immunotherapy and continued immunosuppression to prevent severe rejection and ICI discontinuation. An expert multidisciplinary team is necessary for the shared decision-making process and the optimal management of complex clinical conditions, such as in the case presented here. Large-scale real-world studies are needed to analyze these particular clinical conditions excluded by registrational phase III clinical trials (17).

## Data Availability

The raw data supporting the conclusions of this article will be made available by the authors, without undue reservation.
